# Case Report: developmental delay and intellectual disability linked to a maternally inherited derivative chromosome 3 from a t(3;8) translocation

**DOI:** 10.3389/fgene.2025.1662915

**Published:** 2025-11-27

**Authors:** Andrés León, Alex S. Aguirre, Anna Lindstrand, Marlene Ek, Vanessa I. Romero

**Affiliations:** 1 School of Medicine, Universidad San Francisco de Quito, Quito, Ecuador; 2 Department of Pediatrics, Boston Children’s Hospital, Boston, MA, United States; 3 Department of Clinical Genetics and Genomics, Karolinska University Hospital, Stockholm, Sweden

**Keywords:** derivative chromosomes, WGS, neurodevelopmental disorders, 3p deletion syndrome, 8q duplication, case report, chromosomal rearrangement, structural variation

## Abstract

Chromosomes 3 and 8 harbor genes essential for neurodevelopment, skeletal formation, and metabolic regulation. We report a case of two half-siblings with neurodevelopmental delay and intellectual disability who inherited a derivative chromosome 3 from their asymptomatic mother. Chromosomal microarray analysis first identified a 7.12 Mb deletion in 3p26.3–p26.1 and a 48.86 Mb duplication in 8q22.1–q24.3, and findings were further characterized by whole genome sequencing and manual structural interpretation. The 3p deletion involved four pathogenic genes (*CHL1, CNTN6, CNTN4, ITPR1*) associated with cognitive impairment, ataxia, and motor dysfunction. The 8q duplication affected 50 dosage-sensitive genes implicated in developmental and neurological disorders. Together, these chromosomal imbalances explain the siblings’ phenotype and underscore the contribution of gene dosage effects to neurodevelopmental disease. This case highlights the utility of combining chromosomal microarray and genome sequencing in the diagnosis of complex rearrangements and emphasizes the importance of early genetic counseling and intervention.

## Introduction

Chromosome 3 and chromosome 8 play crucial roles in human development, containing genes essential for neurological, skeletal, and metabolic functions. The short arm of chromosome 3 (3p) harbors genes associated with neurodevelopmental processes, and deletions in this region have been linked to developmental delay, intellectual disability, and congenital anomalies (Shoukier et al., 2013). The long arm of chromosome 8 (8q) harbors genes essential for craniofacial development, skeletal growth, and metabolic regulation. Duplications within this region have been associated with syndromic phenotypes, including intellectual disability and characteristic facial dysmorphism (Hancarova et al., 2013). Understanding the combined impact of genetic rearrangements in cases of neurodevelopmental disorders is crucial as these changes can result in a disruption of the dosage-sensitive genes that are essential for brain development, neuronal connectivity, cognitive function, and motor development (Basilicata and Valsecchi, 2021). The importance of understanding these genetic abnormalities in neurodevelopmental disorders may lead to early diagnosis, early intervention, and genetic counseling for the patients.

Derivative chromosomes (der) result from structural chromosomal rearrangements, including translocations, insertions, or deletions. These aberrations can occur *de novo* or be inherited from a balanced carrier parent, leading to variable clinical manifestations depending on the genes disrupted (Higgins et al., 2020). The phenotypes of derivative chromosomes are influenced by multiple factors, including the size and genomic location of the affected regions, as well as the functional relevance of the altered genes (Zhang et al., 2019). Inherited derivative chromosomes can be particularly challenging to characterize due to their potential to segregate differently in offspring, leading to variable expressivity and incomplete penetrance (Kruszewski et al., 2021). Their identification is crucial in clinical genetics as they correlate with congenital anomalies, developmental delays, and intellectual disabilities.

This is a case of two half-siblings with developmental delay and intellectual disability linked to a maternally inherited derivative chromosome 3, which has not been reported before. ISCN/HGVS description is shown in Table 1. This case underscores the importance of chromosomal microarray analysis in diagnosing neurodevelopmental disorders and guiding clinical management.

**TABLE 1:** ISCN/HGVS of mother and siblings.

Individual	ISCN annotation (GRCh37)	HGVS annotation (GRCh37)	Notes/Interpretation
Mother	seq [GRCh37] der (3)del (3) (pterp26.1)ins (3; 8) (p26.1; qterq24.12)ins (3; 8) (p26.1; q23.3q23.2q)ins (3; 8) (p26.1; q23.1q23.1)ins (3; 8) (p26.1; q22.1q23.1)ins (3; 8) (p26.1; q24.12q24.12)ins (3; 8) (p26.1; q23.1q23.1), der (5)del (5) (q34q34)ins (5; 3) (q34; p26.1p26.1)ins (5; 3) (q34; p26.2p26.2)ins (5; 3) (q34; p26.2p26.2)ins (5; 8) (q34; q24.11q23.3)ins (5; 8) (q34; q24.11q24.11)ins (5; 8) (q34; q24.11q24.11), der (8)del (8) (q22.1qter)ins (8) (p22.1q23.1q23.1)ins (8; 3) (p22.1; p26.2pter)	NC_000003.11:g.pter_7232050delins [NC_000008.10:g.119049296_qterinv; g.109547907_115172261inv; g.109095070_109274778; g.97173775_108683653; g.118533099_119049035; g.108683654_109094620inv] NC_000005.9:g.161588676_161589010delins [NC_000003.11:g.4813706_7230653; g.3317057_3374290; g.3313386_3317057; NC_000008.10:g.115172261_117950918inv; g.118401588_118532987; g.117950918_118400962inv] NC_000008.10:g.97171651_qterdelins [g.109274819_109546541inv; NC_000003.11:g.pter_3313336inv]	Complex chromosomal rearrangement involving chromosomes 3, 5, and 8, including multiple insertions and deletions
Siblings	seq [GRCh37] der (3)del (3) (p26.1)ins (3; 8) (p26.1; qterq24.12)ins (3; 8) (p26.1; q23.3q23.2q)ins (3; 8) (p26.1; q23.1q23.1)ins (3; 8) (p26.1; q22.1q23.1)ins (3; 8) (p26.1; q24.12q24.12)ins (3; 8) (p26.1; q23.1q23.1)mat	NC_000003.11:g.pter_7232050delins [NC_000008.10:g.119049296_qterinv; g.109547907_115172261inv; g.109095070_109274778; g.97173775_108683653; g.118533099_119049035; g.108683654_109094620inv]	Maternally inherited derivative chromosome 3 with 3p26.3–p26.1 deletion and 8q22.1–q24.3 duplication

## Case report

In this case, two siblings were referred to our genetic outpatient clinic with gross motor delay and intellectual disability. The eldest, an 11-year-old female born at 37 weeks, experienced severe neonatal respiratory distress (HP:0006485), requiring a 2-week NICU admission (HP:0002095). She exhibited significant gross motor delay (HP:0001270), sitting at 12 months and walking at 36 months, along with intellectual disability (HP:0001249), diminished strength (HP:0003676, 4/5), left ptosis (HP:0000598), bilateral hypertrichosis (HP:0000990), poor dentition (HP:0000250), and onychomycosis (HP:0002329). The youngest, an 8-year-old male born at 36 weeks, had no perinatal complications but showed a similar developmental pattern, sitting at 9 months and walking at 5 years, with intellectual disability (HP:0001249), diminished strength (HP:0003676, 3/5), poor dentition (HP:0000250), and onychomycosis (HP:0002329). Despite preserved mobility (HP:0002368), neither sibling’s weakness improved with physical therapy. Despite some differences in their motor milestones and strengths, both siblings exhibited similar phenotypic features such as intellectual disability (HP:0001249), diminished strength (HP:0003676), and other developmental delays (HP:0001263). Their mother is asymptomatic, and while they share the same mother, they have different fathers who are not in contact.

## Molecular analysis

Genome-Scan Chromosomal microarray analysis (CMA) was performed for both siblings using different SNP-based array platforms. The siblings were analyzed with a 180K aCGH + SNP array containing ∼180,000 probes (120,000 aCGH probes and 60,000 SNP probes), which enabled the genome-wide detection of copy number variations (CNVs), including large deletions and duplications, as well as unbalanced rearrangements. The findings in the siblings prompted parental testing. The mother was tested using the Invitae Chromosomal Microarray platform, which employs an Illumina SNP array with ∼1.8 million probes, providing tenfold higher probe density and an average resolution of ∼1.5 kb. This platform includes targeted enrichment of over 4,800 clinically relevant genes with high exonic coverage, allowing for more precise detection of smaller CNVs and regions of homozygosity, in addition to improved sensitivity for mosaicism.

These results showed that the mother carried a smaller, non-identical deletion on chromosome 3, but no copy number alterations were found on chromosome 8. This indicated that there may be a more complex rearrangement in the mother, causing the genetic variants in the two children. This in turn led to the decision to perform whole-genome sequencing.

**FIGURE 1:**
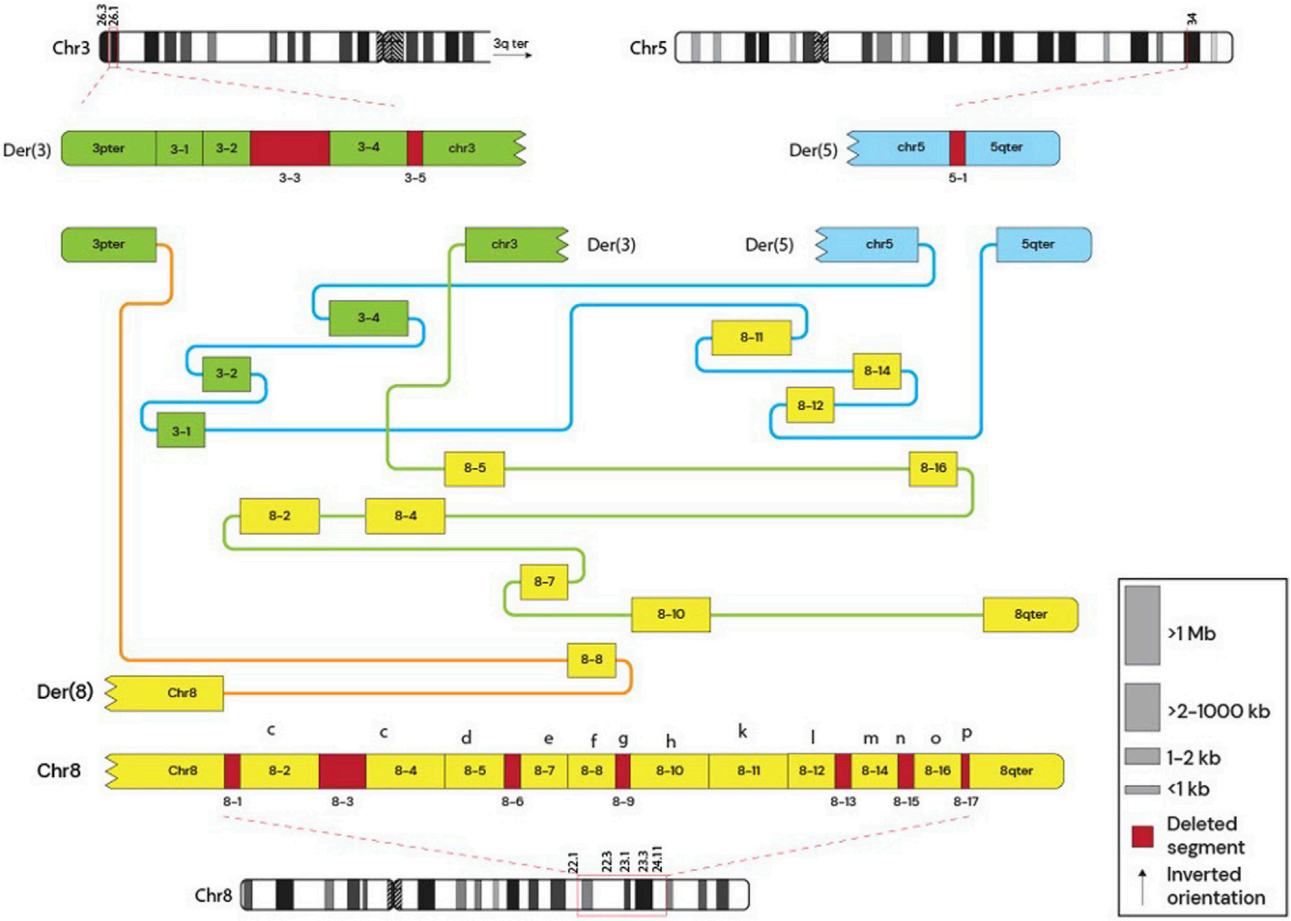
Mother’s Derivative chromosomes. Subway plot of the mother’s derivative chromosomes. The schematic shows deleted regions and the arrangement between different loci. The figure shows that the mother had 3 derivative chromosomes involving a complex rearrangement between chromosomes 3, 5, and 8.

**FIGURE 2:**
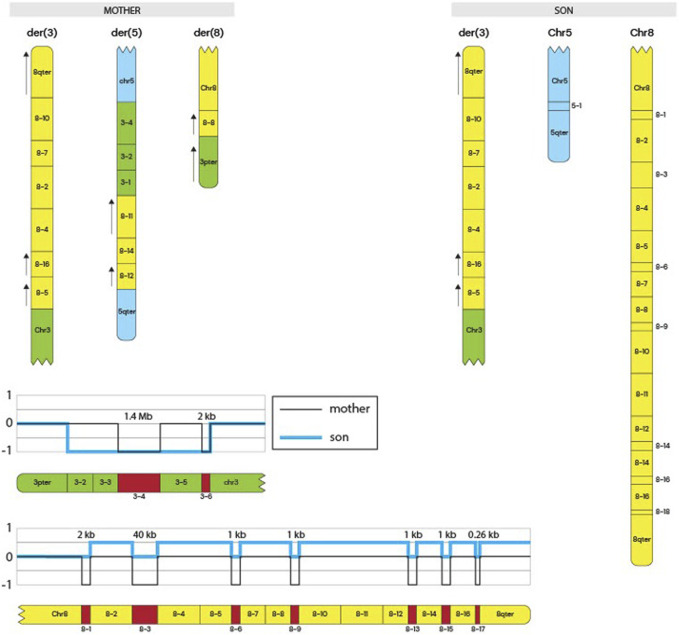
Siblings derivative chromosomes and Copy Number Track. The schematic represents the derivative chromosomes present in the mother and in the siblings (only the son’s schematic shown). It shows the complex rearrangement in the mother and the inheritance of the derivative 3 chromosome to the siblings that led to the symptoms discussed. Copy number tracks are shown at the bottom of the figure, indicating CNVs in both the mother and the siblings.

Whole-genome sequencing was performed for the mother and son to further characterize the structural variants. Genomic DNA was extracted using the *Qiagen Minikit* (Qiagen, Hilden, Germany), and sequencing libraries were prepared with the *Illumina TruSeq DNA PCR-Free kit*. Libraries were sequenced on the *NovaSeq X* platform (Illumina, San Diego, CA, United States). Raw sequencing reads were aligned to the GRCh37 reference genome, and bioinformatic analysis was conducted following the workflow described by Stranneheim et al. (2021). Copy number alterations were manually interpreted using *CytoSure Interpret Software* (Oxford Gene Technology, Oxfordshire, United Kingdom), and the rearrangement structure was delineated using the *Integrative Genomics Viewer (IGV)*. The mother’s derivative chromosome arrangement is shown in Figure 1 and the comparison between the mother and the siblings derivative chromosomes is shown in Figure 2.

**FIGURE 3:**
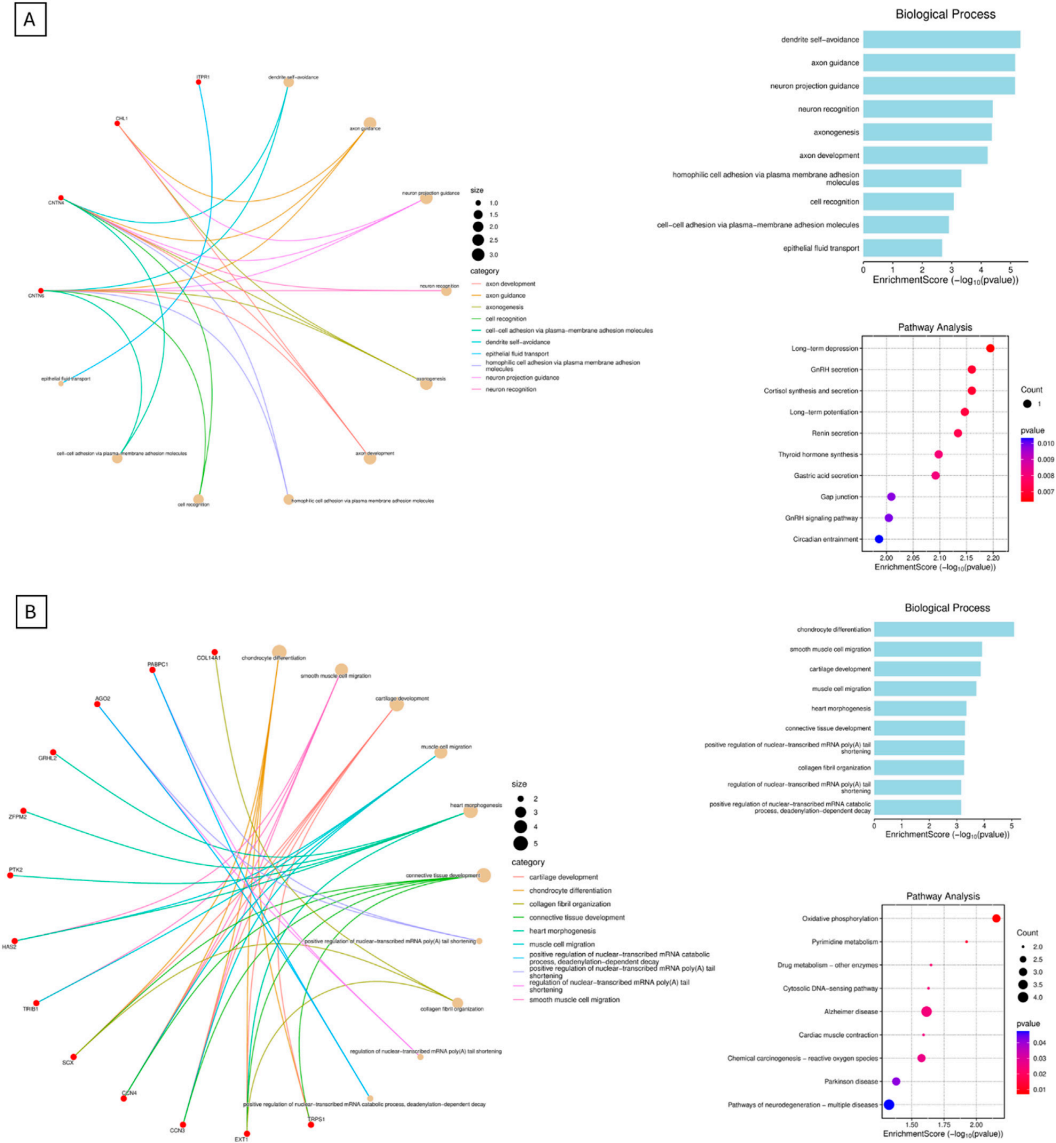
Integrating pathway enrichment. This is the data obtained from the integrating pathway enrichment analysis. The figures show Gene Ontology (GO) categories—biological process (BP), as well as Kyoto Encyclopedia of Genes and Genomes (KEGG) pathways. Cellular component (CC), and molecular function (MF) were not shown. **(A)** shows GO BP and KEGG pathways for derivative chromosome 3, while **(B)** shows GO BP and KEGG for derivative chromosome 8.

Pathway enrichment analysis was performed to investigate the biological significance of the genes located within the deleted chromosomal region (3p26.3–3p26.1). The list of deleted or duplicated genes (Supplementary Table S1,S2) for each chromosome was submitted to SRplot, an online bioinformatics platform that integrates functional annotation with multiple databases. Enrichment was assessed across the Gene Ontology (GO) categories—biological process (BP), cellular component (CC), and molecular function (MF)—as well as Kyoto Encyclopedia of Genes and Genomes (KEGG) pathways. Enrichment results were considered significant based on adjusted *p*-values provided by the tool (Tang et al., 2023). The analysis highlighted pathways and functional clusters related to neurodevelopment, synaptic signaling, and calcium channel regulation, which are consistent with the clinical features observed in the patients. These results are shown in Figure 3.

## Discussion

In this case report, we present the clinical description of a derivative chromosome 3 resulting from a rearrangement between chromosomes 3 and 8 (Figure 2). This rearrangement leads to the deletion of the 3p26.3–3p26.1 region and the duplication of the 8q22.1–8q24.3 region. This is shown in Figure 2, and by comparing CNVs from the mother to the siblings, we can see that they have a greater deletion pattern in the 3p region. Table 1 shows the ISCN/HGVS description, showing the complex triple rearrangement in the mother and the inherited derivative chromosome 3 in the siblings. The molecular analysis revealed a 7.12 Mb deletion in the 3p26.3–3p26.1 region in both siblings, affecting 14 genes, of which four were identified as pathologically significant. These genes are CHL1, CNTN6, CNTN4, and ITPR1, and they are associated with neurodevelopmental disorders, developmental delays, cerebellar ataxia, motor impairment, and diminished muscle strength. The specific function of each gene can be found in the Supplementary Tables S1,S2. The correlation between the function of these genes and the clinical manifestations observed in the siblings is explained by Figure 3. The pathway enrichment analysis showed that the 3p deleted region has a higher impact on neurodevelopmental consequences, as it shows that these genes are heavily related to neurological functions and pathways. On the other hand, 8q duplication showed some neurological association, but also showed enrichment in other pathways that could be related to muscle disorders in the patients. This may explain why the symptomatology is more associated with other cases of 3p deletions like 3p deletion syndrome. This highlights the importance of genetic analysis in patients with complex phenotypes. The siblings also exhibit a 48.86 Mb duplication in the 8q22.1–8q24.3 region (Supplementary Table S1). This duplication affects 193 genes, of which 50 were identified as being of pathological significance. Figure 3 shows that the selected genes are implicated in neurodevelopmental disorders, motor development delay, and hypertrichosis, all of which are observed in the patients.

These clinical manifestations may result from gene dosage effects, Supplementary Tables S1,S2 show the HI (Haploinsufficiency) scores indicate whether having only one functional copy of a gene can cause a phenotype, while TS (Triplosensitivity) scores indicate if having three copies of a gene (two copies plus an extra one) causes a phenotype, nevertheless, many genes didn’t have information for their HI and TS scores, which shows importance of research involving WGS to understand deeper genetic routes that are being affected. Gene dosage effects is a mechanism that has been implicated in various syndromes. Gene dosage alterations can lead to abnormal symptomatology through mechanisms such as gene product aggregation, degradation, chaperone overload, or insufficient transcription factor availability, all of which have been linked to developmental disorders, including those described in this case (Basilicata and Valsecchi, 2021). In the context of the described chromosomal rearrangement involving a deletion of the 3p26.3-3p26.1 region and duplication of the 8q22.1-8q24.3 region, gene dosage effects are particularly pertinent. The deletion on chromosome 3 results in haploinsufficiency, where a single functional copy of a gene does not produce sufficient gene products for normal function. Similar cases, like the 3p deletion syndrome shown to be autosomal dominant, which explains the penetrance and presentation of symptoms in the siblings. The mother does not have the same phenotype, as it shows a balanced-like translocation in Figure 1. This mechanism has been implicated in several human diseases, as some genes are dosage-sensitive and require a strict level of expression to maintain cellular processes (Rice and McLysaght, 2017). The duplication on chromosome 8 may lead to overexpression of genes, potentially causing detrimental effects due to an imbalance in gene product concentrations. For instance, duplications of the PMP22 gene are known to cause Charcot-Marie-Tooth type 1A disease by increasing gene dosage, leading to peripheral nerve dysfunction (Lupski and Stankiewicz, 2005). Furthermore, gene dosage imbalances can disrupt the formation and function of protein complexes as the relative proportions of subunits become skewed. This disruption can impair cellular networks and lead to dominant phenotypes associated with various genetic disorders (Veitia et al., 2013).

Duplications within the 8q region have been reported previously. For the specific duplication described here, only one other case has been published, in which the main manifestations were cleft lip, palate, and neurodevelopmental disorders; however, that duplication occurred on a derivative chromosome 22 (Rezek et al., 2014). Additional reports of 8q22.2–8q24 duplications have described clinical phenotypes overlapping with those of our patients, including developmental delay and intellectual disability (Concolino et al., 2011) (Supplementary Table S2). Similarly, deletions in the 3p region have been reported in approximately 60 cases of familial del (3p) syndrome, with variable deletion sizes but consistent phenotypes such as ptosis, neurodevelopmental disorders, and motor delay (Fu et al., 2021). One report involving the same 3p26.3–3p26.1 region described overlapping symptoms with our patients, with the expectation that additional features may appear over time (Martins et al., 2021). While these rearrangements have been documented independently, to our knowledge, this is the first report of a single derivative chromosome simultaneously carrying a 3p26.3–p26.1 deletion, and an 8q22.1–q24.3 duplication, and the first time such a rearrangement has been shown to be inherited from an otherwise asymptomatic parent. This highlights the novelty of our case and underscores the importance of comprehensive testing strategies, such as chromosomal microarray and WGS, in detecting cryptic but clinically significant rearrangements. Early identification of these abnormalities can provide critical information for prognosis, genetic counseling, and targeted interventionsConclusion.

## Conclusion

In conclusion, this case highlights the critical role of chromosomal microarray analysis and WGS in diagnosing complex neurodevelopmental disorders caused by structural chromosomal rearrangements. The identification of a maternally inherited derivative chromosome 3, with a deletion of the 3p26.3-3p26.1 region and duplication of the 8q22.1-8q24.3 region, underscores the importance of genetic testing in uncovering the underlying genetic causes of developmental delay and intellectual disability. This report not only expands the understanding of gene dosage effects in the context of chromosomal aberrations but also emphasizes the need for early genetic counseling and intervention in affected families.

## Data Availability

The original contributions presented in the study are included in the article/Supplementary Material, further inquiries can be directed to the corresponding author.
