# Supplementary material for: Case Report: developmental delay and intellectual disability linked to a maternally inherited derivative chromosome 3 from a t(3;8) translocation
Source: Front Genet. 2025 Nov 27;16:1662915. doi: 10.3389/fgene.2025.1662915 (PMC12694934; doi:10.3389/fgene.2025.1662915)
Supplement: Supplementary file 2 [file Supplementaryfile2.docx]

Supplementary Material

# Supplementary Data

Supplementary Material should be uploaded separately on submission. Please include any supplementary data, figures, and/or tables.

Supplementary material is not typeset, so please ensure that all information is clearly presented, the appropriate caption is included in the file and not in the manuscript, and that the style conforms to the rest of the article.

# Supplementary Figures and Tables

**Supplementary Tables**

**Supplementary Table 1. Pathogenic genes associated with the 3p deletion in the 3p26.3–3p26.1** **region.**

| **Gene** | **Function** | **Possible Relation to Symptoms** | **HI Score** | **TS Score** | **Reference** |
| --- | --- | --- | --- | --- | --- |
| **CHL1** (Cell Adhesion Molecule L1 Like) | Encodes a neural cell adhesion molecule involved in nervous system development and synaptic plasticity. | Deletion of CHL1 has been associated with intellectual disability, language delay, and impaired motor skills. | 0 (No evidence) | 0 (No evidence) | Loers et al. (2021) |
| **CNTN6** (Contactin 6) | Encodes a member of the immunoglobulin superfamily functioning as a cell adhesion molecule involved in axon connections in the developing nervous system. | Deletions involving CNTN6 have been linked to intellectual disability and developmental delays. | 0 (No evidence) | 0 (No evidence) | Hu et al. (2015) |
| **CNTN4** (Contactin 4) | Encodes a neural cell adhesion molecule implicated in the formation of axon connections. | Deletions of CNTN4 have been associated with intellectual disability and developmental delays. | 0 (No evidence) | 0 (No evidence) | Zheng et al. (2021) |
| **ITPR1** (Inositol 1,4,5-Trisphosphate Receptor Type 1) | Encodes a receptor that mediates the release of intracellular calcium, playing a role in various cellular processes, including synaptic transmission. | Deletions affecting ITPR1 can lead to cerebellar ataxia, which may manifest as motor delays and diminished strength. | 1 (Little Evidence) | 0 (No evidence) | Novak et al. (2010) |

**Supplementary Table 2. Pathogenic genes associated with the 8q duplication in the 8q22.1-8q24.3** **region.**

| **Gene** | **Function** | **Potential Relation to Patient's Symptoms** | **HI Score** | **TS Score** | **Reference** |
| --- | --- | --- | --- | --- | --- |
| **TRAPPC9** | Involved in neuronal development; mutations linked to intellectual disability and motor delay. | Intellectual disability and motor delays observed in the patient may be associated with TRAPPC9 dysfunction. | 30 (autosomal recesive) | 0 (No evidence) | Wilton et al. (2020) |
| **RAD21** | Plays a role in chromosome cohesion; mutations associated with Cornelia de Lange syndrome, characterized by ptosis, hypertrichosis, and developmental delays. | The patient's ptosis, hypertrichosis, and developmental delays could be linked to RAD21 anomalies. | 3 (sufficient evidence) | 0 (No evidence) | Kiefer et al. (2023) |
| **PTK2** | Encodes focal adhesion kinase, important for muscle development and function. | Diminished muscle strength (4/5) in the patient might be related to PTK2 abnormalities. | N/A | N/A | Di Gregorio et al. (2013) |
| **AGO2** | Essential component of the RNA-induced silencing complex; involved in gene silencing and regulation. | Neurodevelopmental defects in the patient could be associated with AGO2 dysfunction. | N/A | N/A | Lessel et al. (2020) |
| **NDRG1** | Involved in Schwann cell differentiation and myelin sheath maintenance; mutations cause Charcot-Marie-Tooth disease, leading to weakness and motor delay. | The patient's motor delays and muscle weakness may be linked to NDRG1 anomalies. | 30 (autosomal recesive) | 0 (No evidence) | Marechal et al. (2021) |
| **KCNQ3** | Encodes a potassium channel subunit; mutations associated with epilepsy and intellectual disability. | Intellectual disability observed in the patient could be related to KCNQ3 dysfunction. | N/A | N/A | Edmond et al. (2024) |
| **TRPS1** | Transcription factor; mutations cause Tricho-Rhino-Phalangeal Syndrome, characterized by dental anomalies and hypertrichosis. | The patient's dental issues and hypertrichosis may be associated with TRPS1 anomalies. | 3 (sufficient evidence) | 0 (No evidence) | Chen et al. (2010) |
| **SLC39A4** | Zinc transporter; mutations lead to acrodermatitis enteropathica, affecting skin, nails, and teeth. | Poor dentition and onychomycosis in the patient could be linked to SLC39A4 dysfunction. | N/A | N/A | Zhong et al. (2020) |
| **EXT1** | Involved in heparan sulfate biosynthesis; mutations cause hereditary multiple exostoses, linked to skeletal abnormalities and potential gross motor delays. | The patient's gross motor delays might be related to EXT1 anomalies. | 3 (sufficient evidence) | 0 (No evidence) | Pacifi (2017) |
| **UQCRB** | Component of mitochondrial complex III; mutations can lead to mitochondrial disorders, causing muscle weakness and motor delays. | Muscle weakness and motor delays in the patient may be associated with UQCRB dysfunction. | N/A | N/A | Hock et al. (2020) |
| **SLC30A8** | Zinc transporter involved in insulin secretion; mutations associated with glucose metabolism disorders. | While not directly linked to the patient's symptoms, metabolic components cannot be ruled out. | N/A | N/A | Uddin et al. (2024) |
| **KCNK9** | Encodes a potassium channel; mutations cause Birk-Barel syndrome, characterized by intellectual disability and hypotonia. | Intellectual disability and diminished muscle tone in the patient could be linked to KCNK9 anomalies. | 0 (No evidence) | 0 (No evidence) | Cousin et al. (2024) |
| **RIMS2** | Regulates synaptic vesicle exocytosis; mutations associated with intellectual disability. | Intellectual disability observed in the patient may be related to RIMS2 dysfunction. | N/A | N/A | Mechaussier et al. (2020) |
| **FBXO32** | Encodes a muscle-specific ubiquitin ligase; involved in muscle atrophy. | Muscle weakness in the patient could be associated with FBXO32 anomalies. | N/A | N/A | Usui et al. (2011) |
| **CCN3** | Involved in skeletal development; mutations linked to skeletal abnormalities and motor issues. | The patient's motor issues and skeletal abnormalities might be related to CCN3 dysfunction. | N/A | N/A | Matushita et al. (2013) |
| **DCAF13** | Associated with developmental syndromes and neurodevelopment. | Developmental delays in the patient could be linked to DCAF13 dysfunction. | N/A | N/A | Manzoor et al. (2023) |
| **ZFPM2** | Essential for lung development; mutations cause congenital diaphragmatic hernia and neonatal respiratory distress. | The patient's neonatal respiratory distress may be related to ZFPM2 dysfunction. | 1 (Little Evidence) | 0 (No evidence) | Longoni et al. (2014) |
| **COL14A1** | Encodes collagen XIV; associated with connective tissue integrity and muscle function. | Connective tissue abnormalities may contribute to muscle weakness in the patient. | N/A | N/A | Ansorge et al. (2009) |
| **TSPYL5** | Involved in neuronal development; mutations linked to neuroblastoma. | The patient's intellectual disability and developmental delays may be related to TSPYL5 dysfunction. | N/A | N/A | Stainczyk & Westermann. (2021) |
| **EMC2** | Involved in neurological disorders; essential for synaptic function. | The patient's intellectual disability and neurological issues could be associated with EMC2 anomalies. | N/A | N/A | Ren et al. (2022) |
| **PABPC1** | Involved in synaptic function and brain development. | Could contribute to intellectual disability and neurodevelopmental delays. | N/A | N/A | Wegler et al. (2022) |
| **YWHAZ** | Plays a key role in neuronal signaling and neurodevelopment. | May be linked to intellectual disability and developmental delay. | N/A | N/A | Antón-Galindo et al. (2022) |
| **GRHL2** | Involved in craniofacial development. | Could be associated with ptosis observed in the patient. | N/A | N/A | De Vries et al. (2021) |
| **NCALD** | Plays a role in neurological phenotypes. | May contribute to intellectual disability and neurodevelopmental issues. | N/A | N/A | Upadhyay et al. (2019) |
| **RRM2B** | Involved in mitochondrial function and muscle maintenance. | Mitochondrial dysfunction may explain muscle weakness. | N/A | N/A | Keshavan et al. (2019) |
| **UBR5** | Associated with neurodevelopmental syndromes. | May contribute to intellectual disability and motor delays. | N/A | N/A | Sabeh et al. (2024) |
| **ODF1** | Plays a role in sperm development. | Limited direct link, but could contribute to sperm development | N/A | N/A | Hetherington et al. (2012) |
| **KLF10** | Regulates skeletal and muscular function. | May contribute to muscle weakness and motor delay. | N/A | N/A | Kammoun et al. (2021) |
| **ATP6V1C1** | Mitochondrial function and muscle maintenance. | Dysfunction may contribute to muscle weakness. | N/A | N/A | Fairley et al. (2023) |
| **BAALC** | Involved in brain development. | Could be associated with intellectual disability. | N/A | N/A | Tanner et al. (2001) |
| **DPYS** | Linked to metabolic disorders. | May explain poor dental health through metabolic dysfunction. | 30 (autosomal recesive) | N/A | Mirzaei et al. (2020) |
| **RGS22** | Involved in neurological signaling pathways. | Could be associated with neurodevelopmental symptoms. | N/A | N/A | Pang et al. (2024) |
| **POLR2K** | Encodes a subunit of RNA polymerase II, affecting gene transcription. | May impact brain function and contribute to intellectual disability. | N/A | N/A | Haijes et al. (2019) |
| **RNF19A** | Involved in neurodegenerative disorders. | May contribute to neurological dysfunction and intellectual disability. | N/A | N/A | Park et al. (2015) |
| **COX6C** | Mitochondrial gene involved in oxidative phosphorylation. | Dysfunction may contribute to muscle weakness. | N/A | N/A | Wang et al. (2022) |
| **EIF3H** | Plays a role in translation regulation and brain development. | Could contribute to intellectual disability. | N/A | N/A | Choudhuri et al. (2013) |
| **MATN2** | Involved in skeletal and muscular development. | Could contribute to gross motor delay. | N/A | N/A | Deák et al. (2014) |
| **FZD6** | Regulates craniofacial and ectodermal development. | Could be linked to ptosis and facial anomalies. | N/A | N/A | Mohammadi-Asl et al. (2017) |
| **CCN4** | Regulates skeletal formation and chondrocyte differentiation. | May be linked to skeletal abnormalities contributing to motor delay. | N/A | N/A | Ono et al. (2010) |
| **CSMD3** | Implicated in neurological disorders. | May be linked to intellectual disability. | N/A | N/A | Song et al. (2022) |
| **ZHX2** | Involved in brain function and neuronal development. | May contribute to intellectual disability. | N/A | N/A | Kawamura et al. (2018) |
| **ZHX1** | Plays a role in neuronal differentiation and transcriptional regulation. | Could be linked to neurodevelopmental delays. | N/A | N/A | Kim et al. (2007) |
| **SNTB1** | Associated with neuromuscular disorders and synaptic stabilization. | May contribute to muscle weakness and motor delays. | N/A | N/A | Liu et al. (2021) |
| **DERL1** | Involved in ER-associated degradation, muscle maintenance, and stress responses. | Dysfunction may lead to muscle weakness. | N/A | N/A | Cai et al. (2022) |
| **MTSS1** | Implicated in neural crest disorders and cytoskeletal organization. | Possible link to craniofacial anomalies and ptosis. | N/A | N/A | Brown et al. (2018) |
| **TRIB1** | Associated with metabolic conditions and lipid homeostasis. | Metabolic dysfunction could contribute to poor dental health. | N/A | N/A | Soubeyrand et al. (2015) |
| **NAPRT** | Involved in NAD metabolism and neuromuscular disorders. | Potential contributor to muscle weakness and motor delay. | N/A | N/A | Duarte-Pereira et al. (2021) |
| **SCX** | Essential for tendon and muscle function. | Could contribute to motor delay and muscle-related symptoms. | N/A | N/A | Paterson et al. (2020) |
| **GSDMD** | Plays a role in immune response and inflammation. | Could contribute to susceptibility to infections, including onychomycosis. | 0 (No evidence) | 0 (No evidence) | Orning & Fitzgerald. (2019) |
| **HAS2** | Regulates extracellular matrix formation, connective tissue, and developmental processes. | Could be linked to connective tissue anomalies affecting muscle strength and craniofacial development. | N/A | N/A | Kuroda et al. (2001) |

References

Ansorge, H. L., Meng, X., Zhang, G., Veit, G., Sun, M., Klement, J. F., Beason, D. P., Soslowsky, L. J., Koch, M., & Birk, D. E. (2009). Type XIV Collagen regulates fibrillogenesis. Journal of Biological Chemistry, 284(13), 8427–8438. <https://doi.org/10.1074/jbc.m805582200>

Antón-Galindo, E., Vecchia, E. D., Orlandi, J. G., Castro, G, Gualda, E. J., Young, A. M. J., Guasch-Piqueras, M., Arenas, C., Herrera-Úbeda, C., Garcia-Fernàndez, J., Aguado, F., Loza-Alvarez, P., Cormand, B., Norton, W. H. J., & Fernàndez-Castillo, N. (2022). Deficiency of the ywhaz gene, involved in neurodevelopmental disorders, alters brain activity and behaviour in zebrafish. Molecular Psychiatry, 27(9), 3739–3748. <https://doi.org/10.1038/s41380-022-01577-9>

Brown, A. S., Meera, P., Altindag, B., Chopra, R., Perkins, E. M., Paul, S., Scoles, D. R., Tarapore, E., Magri, J., Huang, H., Jackson, M., Shakkottai, V. G., Otis, T. S., Pulst, S. M., Atwood, S. X., & Oro, A. E. (2018). MTSS1/Src family kinase dysregulation underlies multiple inherited ataxias. Proceedings of the National Academy of Sciences, 115(52), E12407–E12416. <https://doi.org/10.1073/pnas.1816177115>

Cai, Y., Xu, K., Aihaiti, Y., Li, Z., Yuan, Q., Xu, J., Zheng, H., Yang, M., Wang, B., Yang, Y., Yang, Y., & Xu, P. (2022). Derlin-1, as a potential early predictive biomarker for nonresponse to infliximab treatment in rheumatoid arthritis, is related to autophagy. Frontiers in Immunology, 12, 795912. <https://doi.org/10.3389/fimmu.2021.795912>

Chen, L., Ning, C., & Chao, S. (2010). Mutations in TRPS1 gene in trichorhinophalangeal syndrome type I in Asian patients. British Journal of Dermatology, 163(2), 416–419. <https://doi.org/10.1111/j.1365-2133.2010.09802.x>

Choudhuri, A., Maitra, U., & Evans, T. (2013). Translation initiation factor eIF3h targets specific transcripts to polysomes during embryogenesis. Proceedings of the National Academy of Sciences, 110(24), 9818–9823. <https://doi.org/10.1073/pnas.1302934110>

Cousin, M. A., Veale, E. L., Dsouza, N. R., Tripathi, S., Holden, R. G., Arelin, M., Beek, G., Bekheirnia, M. R., Beygo, J., Bhambhani, V., Bialer, M., Bigoni, S., Boelman, C., Carmichael, J., Courtin, T., Cogne, B., Dabaj, I., Doummar, D., Fazilleau, L., . . . Klee, E. W. (2022). Gain and loss of TASK3 channel function and its regulation by novel variation cause KCNK9 imprinting syndrome. Genome Medicine, 14(1), 62. <https://doi.org/10.1186/s13073-022-01064-4>

De Vries, M., Owens, H. G., Carpinelli, M. R., Partridge, D., Kersbergen, A., Sutherland, K. D., Auden, A., Anderson, P. J., Jane, S. M., & Dworkin, S. (2021). Delineating the roles of Grhl2 in craniofacial development through tissue‐specific conditional deletion and epistasis approaches in mouse. Developmental Dynamics, 250(8), 1191–1209. <https://doi.org/10.1002/dvdy.322>

Deák, F., Mátés, L., Korpos, É., Zvara, Á., Szénási, T., Kiricsi, M., Mendler, L., Keller-Pintér, A., Ózsvári, B., Juhász, H., Sorokin, L., Dux, L., Mermod, N., Puskás, L. G., & Kiss, I. (2014). Extracellular matrilin-2 deposition controls the myogenic program timing during muscle regeneration. Journal of Cell Science, 127(Pt 15), 3240–3256. <https://doi.org/10.1242/jcs.141556>

Di Gregorio, E., Bianchi, F. T., Schiavi, A., Chiotto, A. M. A., Rolando, M., Di Cantogno, L. V., Grosso, E., Cavalieri, S., Calcia, A., Lacerenza, D., Zuffardi, O., Retta, S. F., Stevanin, G., Marelli, C., Durr, A., Forlani, S., Chelly, J., Montarolo, F., Tempia, F., . . . Brusco, A. (2013). A de novo X;8 translocation creates a PTK2-THOC2 gene fusion with THOC2 expression knockdown in a patient with psychomotor retardation and congenital cerebellar hypoplasia. Journal of Medical Genetics, 50(8), 543–551. <https://doi.org/10.1136/jmedgenet-2013-101542>

Duarte-Pereira, S., Fajarda, O., Matos, S., Oliveira, J. L., & Silva, R. M. (2021). NAPRT Expression Regulation Mechanisms: Novel functions predicted by a Bioinformatics approach. Genes, 12(12), 2022. <https://doi.org/10.3390/genes12122022>

Edmond, M. A., Hinojo-Perez, A., Efrem, M., Yi-Chun, L., Shams, I., Hayoz, S., De La Cruz, A., Rodriguez, M. E. P., Diaz-Solares, M., Dykxhoorn, D. M., Luo, Y. L., & Barro-Soria, R. (2024). Lipophilic compounds restore function to neurodevelopmental-associated KCNQ3 mutations. Communications Biology, 7(1), 1181. <https://doi.org/10.1038/s42003-024-06873-4>

Fairley, L. H., Lai, K. O., Wong, J. H., Chong, W. J., Vincent, A. S., D’Agostino, G., Wu, X., Naik, R. R., Jayaraman, A., Langley, S. R., Ruedl, C., & Barron, A. M. (2023). Mitochondrial control of microglial phagocytosis by the translocator protein and hexokinase 2 in Alzheimer’s disease. Proceedings of the National Academy of Sciences, 120(8), e2209177120. <https://doi.org/10.1073/pnas.2209177120>

Haijes, H. A., Koster, M. J., Rehmann, H., Li, D., Hakonarson, H., Cappuccio, G., Hancarova, M., Lehalle, D., Reardon, W., Schaefer, G. B., Lehman, A., Van De Laar, I. M., Tesselaar, C. D., Turner, C., Goldenberg, A., Patrier, S., Thevenon, J., Pinelli, M., Brunetti-Pierri, N., . . . Van Hasselt, P. M. (2019). De Novo Heterozygous POLR2A Variants Cause a Neurodevelopmental Syndrome with Profound Infantile-Onset Hypotonia. The American Journal of Human Genetics, 105(2), 283–301. <https://doi.org/10.1016/j.ajhg.2019.06.016>

Hetherington, L., Schneider, E. K., Scott, C., DeKretser, D., Muller, C. H., Hondermarck, H., Velkov, T., & Baker, M. A. (2016). Deficiency in outer dense fiber 1 is a marker and potential driver of idiopathic male infertility. Molecular & Cellular Proteomics, 15(12), 3685–3693. <https://doi.org/10.1074/mcp.m116.060343>

Hock, D. H., Robinson, D. R. L., & Stroud, D. A. (2020). Blackout in the powerhouse: clinical phenotypes associated with defects in the assembly of OXPHOS complexes and the mitoribosome. Biochemical Journal, 477(21), 4085–4132. <https://doi.org/10.1042/bcj20190767>

Hu, J., Liao, J., Sathanoori, M., Kochmar, S., Sebastian, J., Yatsenko, S. A., & Surti, U. (2015). CNTN6 copy number variations in 14 patients: a possible candidate gene for neurodevelopmental and neuropsychiatric disorders. Journal of Neurodevelopmental Disorders, 7(1), 26. <https://doi.org/10.1186/s11689-015-9122-9>

Kammoun, M., Pouletaut, P., Morandat, S., Subramaniam, M., Hawse, J. R., & Bensamoun, S. F. (2021). Krüppel‐like factor 10 regulates the contractile properties of skeletal muscle fibers in mice. Muscle & Nerve, 64(6), 765–769. <https://doi.org/10.1002/mus.27412>

Kawamura, Y., Yamanaka, K., Poh, B., Kuribayashi, H., Koso, H., & Watanabe, S. (2018). The role of Zhx2 transcription factor in bipolar cell differentiation during mouse retinal development. Biochemical and Biophysical Research Communications, 503(4), 3023–3030. <https://doi.org/10.1016/j.bbrc.2018.08.088>

Keshavan, N., Abdenur, J., Anderson, G., Assouline, Z, Barcia, G., Bouhikbar, L., Chakrapani, A., Cleary, M., Cohen, M. C., Feillet, F., Fratter, C., Hauser, N., Jacques, T., Lam, A., McCullagh, H., Phadke, R., Rötig, A., Sharrard, M., Simon, M., . . . Rahman, S. (2019). The natural history of infantile mitochondrial DNA depletion syndrome due to RRM2B deficiency. Genetics in Medicine, 22(1), 199–209. <https://doi.org/10.1038/s41436-019-0613-z>

Kiefer, L., Chiosso, A., Langen, J., Buckley, A., Gaudin, S., Rajkumar, S. M., Servito, G. I. F., Cha, E. S., Vijay, A., Yeung, A., Horta, A., Mui, M. H., & Canzio, D. (2023). WAPL functions as a rheostat of Protocadherin isoform diversity that controls neural wiring. Science, 380(6651), eadf8440. <https://doi.org/10.1126/science.adf8440>

Kim, S., Park, J., Choi, M., Kim, H., Park, J., Jung, Y., Lee, J., Oh, D., Im, S., Bang, Y., & Kim, T. (2007). Zinc-fingers and homeoboxes 1 (ZHX1) binds DNA methyltransferase (DNMT) 3B to enhance DNMT3B-mediated transcriptional repression. Biochemical and Biophysical Research Communications, 355(2), 318–323. <https://doi.org/10.1016/j.bbrc.2007.01.187>

Kuroda, K., Utani, A., Hamasaki, Y., & Shinkai, H. (2001). Up-regulation of putative hyaluronan synthase mRNA by basic fibroblast growth factor and insulin-like growth factor-1 in human skin fibroblasts. Journal of Dermatological Science, 26(2), 156–160. <https://doi.org/10.1016/s0923-1811(00)00155-9>

Lessel, D., Zeitler, D. M., Reijnders, M. R. F., Kazantsev, A., Nia, F. H., Bartholomäus, A., Martens, V., Bruckmann, A., Graus, V., McConkie-Rosell, A., McDonald, M., Lozic, B., Tan, E., Gerkes, E., Johannsen, J., Denecke, J., Telegrafi, A., Zonneveld-Huijssoon, E., Lemmink, H. H., . . . Kreienkamp, H. (2020). Germline AGO2 mutations impair RNA interference and human neurological development. Nature Communications, 11(1), 5797. <https://doi.org/10.1038/s41467-020-19572-5>

Liu, L., Chen, Y., Lin, X., Wu, M., Li, J., Xie, Q., Sferra, T. J., Han, Y., Liu, H., Cao, L., Yao, M., Peng, J., & Shen, A. (2021). Upregulation of SNTB1 correlates with poor prognosis and promotes cell growth by negative regulating PKN2 in colorectal cancer. pmc.ncbi.nlm.nih.gov. <https://doi.org/10.1186/s12935-021-02246-7>

Loers, G., Kleene, R., Granato, V., Bork, U., & Schachner, M. (2021). Interplay in neural functions of cell adhesion molecule close homolog of L1 (CHL1) and programmed cell death 6 (PDCD6). FASEB BioAdvances. <https://doi.org/10.1096/fba.2021-00027>

Longoni, M., Russell, M., High, F., Darvishi, K., Maalouf, F., Kashani, A., Tracy, A., Coletti, C., Loscertales, M., Lage, K., Ackerman, K., Woods, S., Ward‐Melver, C., Andrews, D., Lee, C., Pober, B., & Donahoe, P. (2014). Prevalence and penetrance of ZFPM2 mutations and deletions causing congenital diaphragmatic hernia. Clinical Genetics, 87(4), 362–367. <https://doi.org/10.1111/cge.12395>

Marechal, D., Dansu, D. K., Castro, K., Patzig, J., Magri, L., Inbar, B., Gacias, M., Moyon, S., & Casaccia, P. (2021). N‐myc downstream regulated family member 1 (NDRG1) is enriched in myelinating oligodendrocytes and impacts myelin degradation in response to demyelination. Glia, 70(2), 321–336. <https://doi.org/10.1002/glia.24108>

Matsushita, Y., Sakamoto, K., Tamamura, Y., Shibata, Y., Minamizato, T., Kihara, T., Ito, M., Katsube, K., Hiraoka, S., Koseki, H., Harada, K., & Yamaguchi, A. (2013). CCN3 protein participates in bone regeneration as an inhibitory factor. Journal of Biological Chemistry, 288(27), 19973–19985. <https://doi.org/10.1074/jbc.m113.454652>

Manzoor, H., Zahid, H., Emerling, C. A., Kumar, K. R., Hussain, H. M. J., Seo, G. H., Wajid, M., & Naz, S. (2023). A biallelic variant of DCAF13 implicated in a neuromuscular disorder in humans. European Journal of Human Genetics, 31(6), 629–637. <https://doi.org/10.1038/s41431-023-01319-7>

Mechaussier, S., Almoallem, B., Zeitz, C., Van Schil, K., Jeddawi, L., Van Dorpe, J., Rey, A. D., Condroyer, C., Pelle, O., Polak, M., Boddaert, N., Bahi-Buisson, N., Cavallin, M., Bacquet, J., Mouallem-Bézière, A., Zambrowski, O., Sahel, J. A., Audo, I., Kaplan, J., . . . Perrault, I. (2020). Loss of Function of RIMS2 Causes a Syndromic Congenital Cone-Rod Synaptic Disease with Neurodevelopmental and Pancreatic Involvement. The American Journal of Human Genetics, 106(6), 859–871. <https://doi.org/10.1016/j.ajhg.2020.04.018>

Mirzaei, M., Kavosi, A., Sharifzadeh, M., Mahjoub, G., Faghihi, M. A., Habibzadeh, P., & Yavarian, M. (2020). A novel stop-gain mutation in DPYS gene causing Dihidropyrimidinase deficiency, a case report. BMC Medical Genetics, 21(1), 138. <https://doi.org/10.1186/s12881-020-01070-6>

Mohammadi-Asl, J., Pourreza, M. R., Mohammadi, A., Eskandari, A., Mozafar-Jalali, S., & Tabatabaiefar, M. A. (2017). A novel pathogenic variant in the FZD6 gene causes recessive nail dysplasia in a large Iranian kindred. Journal of Dermatological Science, 88(1), 134–138. <https://doi.org/10.1016/j.jdermsci.2017.04.017>

Novak, M. J. U., Sweeney, M. G., Li, A., Treacy, C., Chandrashekar, H., Giunti, P., … Houlden, H. (2010). An ITPR1 gene deletion causes spinocerebellar ataxia 15/16: A genetic, clinical and radiological description. Movement Disorders, 25(13), 2176–2182. <https://doi.org/10.1002/mds.23223>

Ono, M., Inkson, C. A., Kilts, T. M., & Young, M. F. (2010). WISP-1/CCN4 regulates osteogenesis by enhancing BMP-2 activity. Journal of Bone and Mineral Research, 26(1), 193–208. <https://doi.org/10.1002/jbmr.205>

Orning, P., Lien, E., & Fitzgerald, K. A. (2019). Gasdermins and their role in immunity and inflammation. The Journal of Experimental Medicine, 216(11), 2453–2465. <https://doi.org/10.1084/jem.20190545>

Pacifici, M. (2017). Hereditary Multiple Exostoses: New Insights into Pathogenesis, Clinical Complications, and Potential Treatments. Current Osteoporosis Reports, 15(3), 142–152. <https://doi.org/10.1007/s11914-017-0355-2>

Pang, X., Gu, L., Han, Q., Xing, J., Zhao, M., Huang, S., Yi, J., Pan, J., Hong, H., Xue, W., Zhou, X., Su, Z., Zhang, X., Sun, L., Jiang, S., Luo, D., Chen, L., Wang, Z., Yu, Y., . . . Li, T. (2024). RGS22 maintains the physiological function of ependymal cells to prevent hydrocephalus. Science China Life Sciences, 68(2), 441–453. <https://doi.org/10.1007/s11427-024-2720-8>

Park, H., Yang, J., Kim, R., Li, Y., Lee, Y., Lee, C., Park, J., Lee, D., Kim, H., & Kim, E. (2015). Mice lacking the PSD-95–interacting E3 ligase, Dorfin/Rnf19a, display reduced adult neurogenesis, enhanced long-term potentiation and impaired contextual fear conditioning. Scientific Reports, 5(1), 16410. <https://doi.org/10.1038/srep16410>

Paterson, Y., Evans, N., Kan, S., Cribbs, A., Henson, F., & Guest, D. (2020). The transcription factor scleraxis differentially regulates gene expression in tenocytes isolated at different developmental stages. Mechanisms of Development, 163, 103635. <https://doi.org/10.1016/j.mod.2020.103635>

Ren, S., Chen, Y., Wang, L., & Wu, G. (2022). Neuronal ferroptosis after intracerebral hemorrhage. Frontiers in Molecular Biosciences, 9, 966478. <https://doi.org/10.3389/fmolb.2022.966478>

Sabeh, P., Dumas, S. A., Maios, C., Daghar, H., Korzeniowski, M., Rousseau, J., Lines, M., Guerin, A., Millichap, J. J., Landsverk, M., Grebe, T., Lindstrom, K., Strober, J., Mouhoub, T. A., Zweier, C., Steinraths, M., Hebebrand, M., Callewaert, B., Jamra, R. A., . . . Campeau, P. M. (2024). Heterozygous UBR5 variants result in a neurodevelopmental syndrome with developmental delay, autism, and intellectual disability. The American Journal of Human Genetics, 112(1), 75–86. <https://doi.org/10.1016/j.ajhg.2024.11.009>

Song, W., Li, Q., Wang, T., Li, Y., Fan, T., Zhang, J., Wang, Q., Pan, J., Dong, Q., Sun, Z. S., & Wang, Y. (2022). Putative complement control protein CSMD3 dysfunction impairs synaptogenesis and induces neurodevelopmental disorders. Brain Behavior and Immunity, 102, 237–250. <https://doi.org/10.1016/j.bbi.2022.02.027>

Soubeyrand, S., Martinuk, A., Naing, T., Lau, P., & McPherson, R. (2015). Role of Tribbles Pseudokinase 1 (TRIB1) in human hepatocyte metabolism. Biochimica Et Biophysica Acta (BBA) - Molecular Basis of Disease, 1862(2), 223–232. <https://doi.org/10.1016/j.bbadis.2015.12.003>

Stainczyk, S. A., & Westermann, F. (2021). Neuroblastoma—Telomere maintenance, deregulated signaling transduction and beyond. International Journal of Cancer, 150(6), 903–915. <https://doi.org/10.1002/ijc.33839>

Tanner, S. M., Austin, J. L., Leone, G., Rush, L. J., Plass, C., Heinonen, K., Mrózek, K., Sill, H., Knuutila, S., Kolitz, J. E., Archer, K. J., Caligiuri, M. A., Bloomfield, C. D., & De La Chapelle, A. (2001). BAALC, the human member of a novel mammalian neuroectoderm gene lineage, is implicated in hematopoiesis and acute leukemia. Proceedings of the National Academy of Sciences, 98(24), 13901–13906. <https://doi.org/10.1073/pnas.241525498>

Uddin, M. M., Hossain, M. T., Hossain, M. A., Ahsan, A., Shamim, K. H., Hossen, M. A., Rahman, M. S., Rahman, M. H., Ahmed, K., Bui, F. M., & Al-Zahrani, F. A. (2024). Unraveling the potential effects of non-synonymous single nucleotide polymorphisms (nsSNPs) on the Protein structure and function of the human SLC30A8 gene on type 2 diabetes and colorectal cancer: An In silico approach. Heliyon, 10(17), e37280. <https://doi.org/10.1016/j.heliyon.2024.e37280>

Upadhyay, A., Hosseinibarkooie, S., Schneider, S., Kaczmarek, A., Torres-Benito, L., Mendoza-Ferreira, N., Overhoff, M., Rombo, R., Grysko, V., Kye, M. J., Kononenko, N. L., & Wirth, B. (2019). Neurocalcin Delta knockout impairs adult neurogenesis whereas half reduction is not pathological. Frontiers in Molecular Neuroscience, 12, 19. <https://doi.org/10.3389/fnmol.2019.00019>

Usui, S., Maejima, Y., Pain, J., Hong, C., Cho, J., Park, J. Y., Zablocki, D., Tian, B., Glass, D. J., & Sadoshima, J. (2011). Endogenous muscle atrophy F-Box mediates Pressure Overload–Induced Cardiac Hypertrophy through regulation of Nuclear Factor-ΚB. Circulation Research, 109(2), 161–171. <https://doi.org/10.1161/circresaha.110.238717>

Wang, C., Lv, J., Xue, C., Li, J., Liu, Y., Xu, D., Jiang, Y., Jiang, S., Zhu, M., Yang, Y., & Zhang, S. (2022). Novel role of COX6c in the regulation of oxidative phosphorylation and diseases. Cell Death Discovery, 8(1), 336. <https://doi.org/10.1038/s41420-022-01130-1>

Wegler, M., Jia, X., Alders, M., Bouman, A., Chen, J., Duan, X., Lauzon, J. L., Mathijssen, I. B., Sticht, H., Syrbe, S., Tan, S., Guo, H., & Jamra, R. A. (2022). De novo variants in the PABP domain of PABPC1 lead to developmental delay. Genetics in Medicine, 24(8), 1761–1773. <https://doi.org/10.1016/j.gim.2022.04.013>

Wilton, K. M., Gunderson, L. B., Hasadsri, L., Wood, C. P., & Schimmenti, L. A. (2020). Profound intellectual disability caused by homozygous TRAPPC9 pathogenic variant in a man from Malta. Molecular Genetics & Genomic Medicine, 8(5), e1211. <https://doi.org/10.1002/mgg3.1211>

Zheng, Y., & Li, S. (2021). The autism risk gene CNTN4 modulates dendritic spine formation. Journal of Neurodevelopmental Disorders. <https://doi.org/10.1186/s11689-021-09355-1>

Zhong, W., Yang, C., Zhu, L., Huang, Y Q., & Chen, Y F. (2020). Analysis of the relationship between the mutation site of the SLC39A4 gene and acrodermatitis enteropathica by reporting a rare Chinese twin: a case report and review of the literature. BMC Pediatrics, 20(1), 34. <https://doi.org/10.1186/s12887> 020 1942 4
